# Molecular Nanomagnets as Qubits with Embedded Quantum-Error Correction

**DOI:** 10.1021/acs.jpclett.0c02213

**Published:** 2020-09-16

**Authors:** A. Chiesa, E. Macaluso, F. Petiziol, S. Wimberger, P. Santini, S. Carretta

**Affiliations:** † Dipartimento di Scienze Matematiche, Fisiche e Informatiche, Università di Parma, I-43124 Parma, Italy; ‡ UdR Parma, INSTM, I-43124 Parma, Italy; ¶ INFN, Sezione di Milano Bicocca, Gruppo Collegato di Parma, Parma, Italy

## Abstract

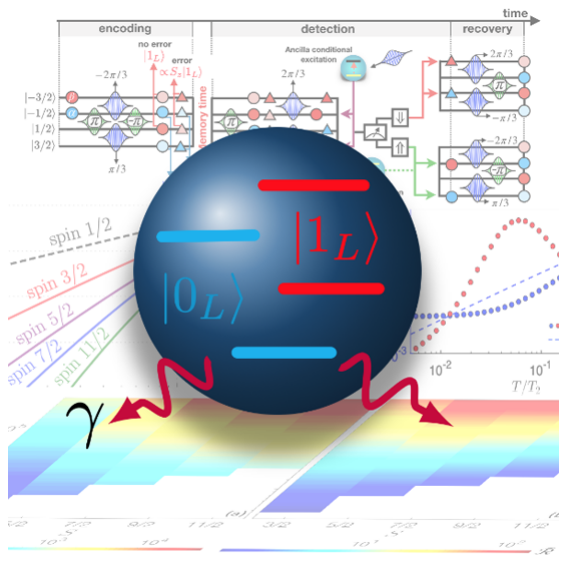

We show that molecular nanomagnets have a potential advantage in the crucial rush toward quantum computers. Indeed, the sizable number of accessible low-energy states of these systems can be exploited to define qubits with embedded quantum error correction. We derive the scheme to achieve this crucial objective and the corresponding sequence of microwave/radiofrequency pulses needed for the error correction procedure. The effectiveness of our approach is shown already with a minimal *S* = 3/2 unit corresponding to an existing molecule, and the scaling to larger spin systems is quantitatively analyzed.

The route toward quantum computers has seen an astonishing boost in the past few years,^1,2^ with noisy intermediate-scale devices^3^ already available to run nontrivial quantum algorithms.^4−9^ However, protecting quantum information from its intrinsic fragility via quantum error correction (QEC) is the striking roadblock that has to be circumvented to really unleash the power of quantum computers.^10^ While non-error-corrected algorithms are based on elementary two-level units called *qubits*, the idea behind QEC is to encode the quantum information into “logical qubits”, objects with more than two possible energy levels. Logical qubits are designed such that errors bring the system in a state outside the computational subspace, making errors in logical qubits detectable and correctable. In standard approaches these extra-states are obtained by encoding a logical qubit into many physical units.^11−18^ However, this makes the practical implementation of QEC and the corresponding quantum computation extremely difficult, because nonlocal quantum gates on a large set of physically distinct objects are needed.^10^ A possible way to overcome this hurdle is by employing a single multilevel quantum object to encode a logical qubit.^19−24^

In this respect, molecular nanomagnets represent the ideal platform, offering many accessible (electronic and nuclear) spin states which could be used to encode a protected qubit. Chemical engineering enabled the realization of molecular systems targeted for specific applications. For instance, careful tailoring of the ligand cage surrounding rare-earth ions enabled the synthesis of bistable single-ion magnets showing high-temperature magnetic hysteresis,^25−30^ thus paving the way to data storage at the single-molecule level. Magnetic molecules were also largely investigated as promising platforms for quantum computation: interesting complexes were designed to meet specific schemes^31−39^ and were chemically optimized to reach very long coherence times.^40−44^ As far as QEC is concerned, one could think of mapping 2^*n*^ molecular levels to *n* qubits^45,48^ and applying standard error-correction codes for independent qubits. However, usually this does not work because real hardware errors in these molecular systems do not typically translate into single-qubit errors, thus making standard codes ineffective.^49^

Conversely, here we show how to encode a single logical qubit into *d* levels of a molecular nanomagnet (*qudit encoding*), endowed with a QEC scheme to protect it against the most harmful errors occurring in molecular qubits, namely, pure dephasing. To this aim, rather than resorting to codes based on abstract generic error models,^20,21,50^ we derive a code which is specific for the class of systems we are considering and hence gives substantially better performance. We introduce error-protected states in such a molecular qudit and design the full sequence of magnetic pulses actually realizing the QEC for generic spin systems. Already existing monomers and dimers can be used to implement our proposal, with an electronic or nuclear spin *S*(34,41,44,46,47,51,52,54) representing the qudit, coupled to a spin 1/2 ancilla, used for error detection.

*Design of QEC Codes for Magnetic Molecules*. To facilitate the implementation of the QEC code, we consider simple molecules described by the Hamiltonian
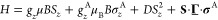
1where *S* is the qudit spin used to embed the error-protected qubit, σ^A^ = 1/2 is an electronic spin, exploited as “ancilla” for error detection, and μ_B_ is the Bohr magneton. This Hamiltonian is realized, e.g., in a complex containing a single magnetic ion with a nuclear spin *S* interacting with its electronic spin or in a dimer consisting of a (molecular) spin *S* weakly coupled to a spin 1/2. In the former case μ is the nuclear magneton (μ_N_), while in the latter μ ≡ μ_B_. The first two terms in eq 1 represent the Zeeman interaction with an external magnetic field *B* along *z*; the third term is the single-ion anisotropy of the qudit (making qudit transitions well energetically distinguishable), and the last term represents a weak exchange or hyperfine ancilla–qudit coupling **Γ**. Because Γ_*x*,*y*_ is much smaller than the difference of qudit and ancilla excitation energies, the eigenstates are simple tensor products of the eigenstates of *S*_*z*_ (|*m*⟩) and σ_*z*_^A^ (|⟩), with *m* = −*S*, ..., *S* and  = ↑, ↓.

The most important error in molecular qubits is given by pure dephasing,^41,43,54^ whose effect can be described by^55^
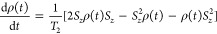
2where *T*_2_ is the dephasing time and ρ(*t*) is the density operator of the qudit. [An analogous term for the ancilla *A* makes a negligible effect, because *A* is practically always in the ground state (see below).] Conversely, spin relaxation is usually very slow at low temperature in these systems, with electronic relaxation times reaching ∼10^2^ ms.^53,54^ Hence, our aim is finding how to use qudit states to correct for the unwanted decoherence due to eq 2. As shown in the Supporting Information, for small *t*/*T*_2_ it is possible to perform the perturbative expansion , with the *error operators*
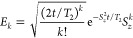
3

This shows that at short *t*/*T*_2_ only low powers of *S*_*z*_ affect the dynamics. By considering the matrix elements of *S*_*z*_^*n*^ we thus define protected qubit states (*code words*) and a QEC procedure to recover the effects of pure dephasing (up to a given order in (*t*/*T*_2_)^*n*^). Inspired by binomial codes on bosonic systems,^49,56^ we introduce protected code words:
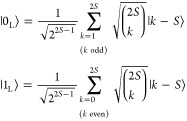
4Here, the summation for |0_L_⟩ (|1_L_⟩) includes only odd (even) *k* in the range [0, 2*S*]. Note that for correcting dephasing up to order (*t*/*T*_2_)^*n*^ (for integer *n*) one needs at least 2*n* levels (see the Supporting Information). Hence, using an integer spin *S*_int_ is admissible, but it does not lead to a better performance as compared to *S*_int_ – 1/2. We thus focus here on half-integer spins. As we show in the Supporting Information, these code words ensure that (*i*) *E*_*k*_ errors bring |0_L_⟩ and |1_L_⟩ to distinguishable (orthogonal) states and (*ii*) the coefficients α and β of a generic superposition of the logical states (α|0_L_⟩ + β|1_L_⟩) are preserved. Therefore, errors are detectable, for (*i*), and correctable, for (*ii*).^57^ We quantify the *ideal* performance of this code by computing the fidelity^58^ of the corrected state ρ_C_(*t*), , i.e., how much the corrected state matches the initial state |ψ_0_⟩ = α|0_L_⟩ + β|1_L_⟩. The final error (probability of finding the system in the wrong state)  is reported in Figure 1 for different values (colors) of the qudit spin *S*. A comparison with a spin 1/2 without QEC (dashed line), shows a remarkable reduction of the error  with our scheme, evidenced also by the gain factor , reported in the inset. Therefore, our QEC scheme is very effective in correcting decoherence, and the residual error decreases by using more levels, i.e., by increasing *S*.

**Figure 1:**
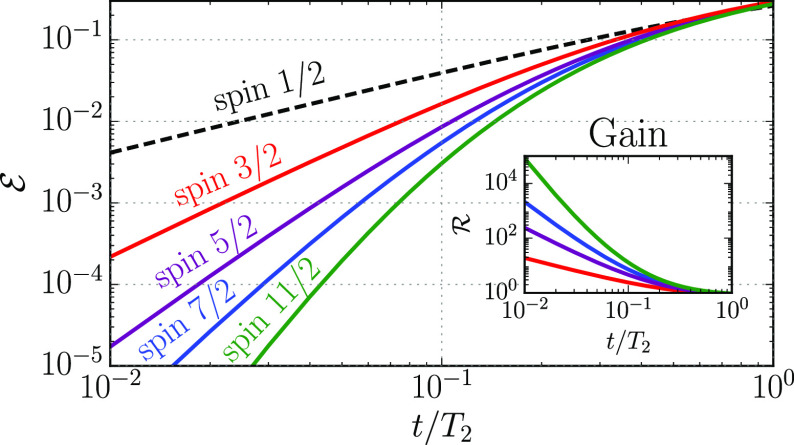
Ideal performance of spin-binomial codes on a spin *S* system, initialized in the pure state , corresponding to the most error prone qubit state.^58^ Colors refer to different values of *S*. The dashed line indicates the error for an uncorrected spin 1/2, i.e. . Inset: gain ratio .

Simple candidates to implement the scheme are molecular complexes consisting of (i) a single magnetic ion coupled to a magnetic nucleus or of (ii) pairs of electronic spins linked by exchange interactions. Several compounds belonging to class (i) exist, with nuclear spins ranging from 3/2 to 7/2^34,41,44,50,54,59^ and remarkable electronic coherence. As far as electronic spins (ii) are concerned, Gd complexes (such as the one reported in ref (45)) can provide an *S* = 7/2 qudit and can also be arranged in dissymmetric, weakly interacting and individually addressable dimers.^46^ Additional levels of the ancilla (not strictly needed in our QEC scheme) can be exploited as an additional resource. A simpler implementation is given by a CrYb electronic spin dimer,^47^ where the effective Yb^3+^ spin doublet provides the ancilla and Cr^3+^ the 4-level qudit.

*Pulse Sequence*. The implementation of this QEC code requires translating the abstract operations above into precise experimental steps. To achieve this, we design for a generic spin *S* a complete sequence of resonant microwave/radiofrequency pulses inducing Δ*m* = ±1 transitions. Figure 2a illustrates it for the minimal *S* = 3/2 case, while the general procedure is detailed in the Supporting Information. Starting with the qudit in a state |ψ(0)⟩ = α|−1/2⟩ + β|−3/2⟩, *encoding* in the protected states is achieved using the four pulses in the left part of the figure. After free evolution of the system (*memory time*), the error *detection* procedure is applied. With *S* = 3/2 only *S*_*z*_ errors can be corrected, and hence, we need to distinguish between the *no-error* and *S*_*z*_*error* cases. To detect the possible error, we excite the ancilla only if no *S*_*z*_ error has occurred, bringing the code words |0_L_/1_L_⟩ to error words ∼*S*_*z*_|0_L_/1_L_⟩. This is achieved by first applying pulses that bring the correct state |ψ(0)⟩ to α|3/2⟩ + β|−1/2⟩, and the state corresponding to an error to α|1/2⟩ + β|−3/2⟩. Then, two simultaneous π pulses are employed to excite the ancilla only if the qudit is in |*m* = −1/2, 3/2⟩ (thanks to the ancilla-qudit coupling Γ). A subsequent measurement of the ancilla projects either into the *no error* (if we find  = ↑) or to the *error* (for  = ↓) subspaces, thus allowing us to detect the error and apply the corresponding recovery procedure to restore |ψ_0_⟩.

**Figure 2:**
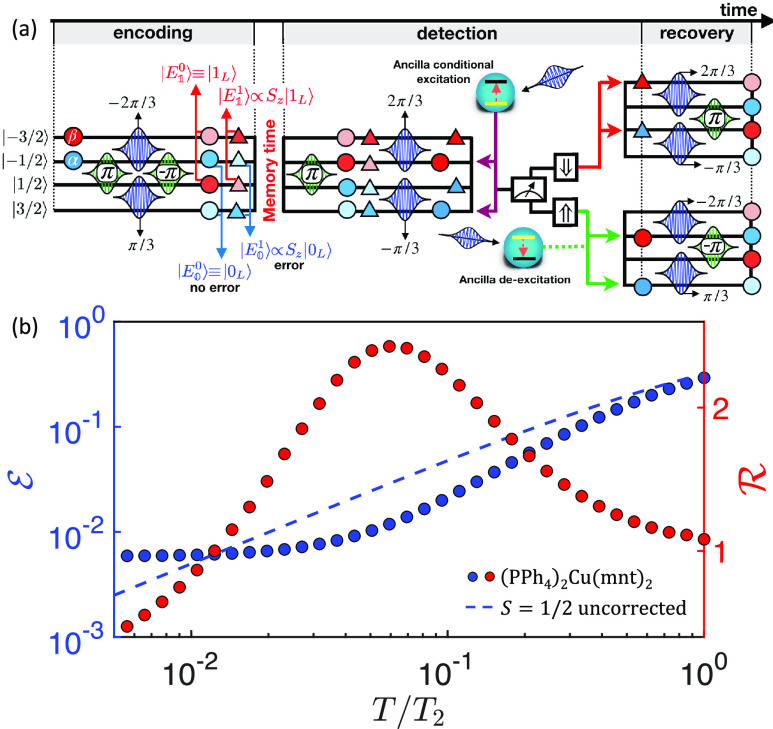
(a) Sequence of pulses implementing the QEC code on *S* = 3/2. Horizontal lines represent the eigenstates of the qudit Hamiltonian, labeled by the corresponding *S*_*z*_ eigenvalue. Time increases from left to right. Magnetic pulses resonant with Δ*m* = ±1 transitions are depicted by Gaussian-shaped peak functions between the two involved levels with the rotation angle θ indicated. Blue (red) symbols represent |0_L_/1_L_⟩ (*S*_*z*_|0_L_/1_L_⟩) states, with color intensity proportional to the modulus of the component and symbol shape changing from the code word to the error word. After encoding, the system evolves freely during the *memory time*. The *detection* pulses are applied, and then a conditional excitation of the ancilla (depending on qudit state |*m*⟩) allows us to detect errors by measuring the ancilla. Finally, depending on the outcome ↑/↓, a different sequence of pulses is applied to recover the encoded state. (b) Simulated final error  (blue circles) as a function of memory time *T* in units of the qudit *T*_2_ and corresponding gain  (red), for the nuclear *S* = 3/2 ^63^Cu qudit in the (PPh_4_)_2_[Cu(mnt)_2_] complex. This is compared with the case of uncorrected spin 1/2 (dashed line).

Larger spin qudits allow us to correct more *E*_*k*_ errors, corresponding to larger powers of *S*_*z*_. To do this, we need to distinguish different errors. This is achieved by mapping each code word and error word to a well-defined |*m*⟩ state. Then we perform a series of conditional excitations and measurements of the two-level ancilla until  = ↑ is found and the corresponding error identified. The series of measurements starts from the most probable errors (corresponding to lower *k* and lower powers of *S*_*z*_). Details on the general procedure for larger spin *S* are given in the Supporting Information.

*Physical Implementation*. General requirements to implement our scheme are the following: (i) Γ_*x*,*y*_ much smaller than the difference between excitation energies of qudit (Δ*m* = ±1) and ancilla (for nondemolition readout); (ii) significant Γ_*z*_, to enable selective excitation of the ancilla depending on the |*m*⟩ state of the qudit and hence error detection. These conditions can be fulfilled by applying a sizable magnetic field. The latter can lead to |*m*⟩|⟩ eigenstates also in the presence of non-negligible transverse anisotropy in eq 1. A suitable system to test the code is the (PPh_4_)_2_[Cu(mnt)_2_] complex reported in ref (54), consisting of a *S* = 3/2 nuclear qudit hyperfine-coupled to an electronic spin. This directly implements the scheme depicted in Figure 2a. To assess the performance of the QEC code, we perform numerical simulations on this existing molecular system by solving the Lindblad equation (eq 2) including continuous dephasing on both qudit and ancilla. The system is subject to pure dephasing in all the steps (including encoding, detection and correction). The molecule is characterized by Γ = (118, 118, 500) MHz, *g*_*z*_^A^ = 2.09, while for the nuclear quadrupole term we assume *D* = 50 MHz, reasonable for ^63^Cu.^60,61^ Given the long *T*_2_^A^ ≈ 10^–1^ ms shown by its electronic ancilla,^54^ for the nuclear qudit we conservatively assume *T*_2_ = 1 ms. In a typical X-band field of 0.3 T, ancilla-qudit states are practically factorized and Γ_z_ = 500 MHz allows us to resolve all transitions using an oscillating field of amplitude 50 G. Figure 2b shows the resulting error probability  as a function of the memory time *T* in units of *T*_2_. This is only slightly affected by *T*_2_^A^, which is brought to a superposition only during fast (electronic) excitations used for error detection (see the Supporting Information), while it is kept in its ground state for the rest of the time. For this reason, we can neglect spin relaxation on the electronic ancilla.^54^

Differently from the ideal case in Figure 1, present simulations include errors due to the finite time *T*_QEC_ required to implement the QEC code, making pure dephasing effective also during this step, as well as imperfect pulses (due to finite bandwidth of the Gaussian pulses) yielding gate errors. It is worth noting that our QEC scheme works well even in these realistic conditions, as shown in Figure 2. Even by using only a 4-level qudit, the performance of the code is remarkable, reducing the final error by a factor ∼2.5 at its maximum. We can define the *optimal working point T̃* as the value of *T* corresponding to the maximum () of , because it represents the ideal time interval before repeating the QEC procedure. In this case we find *T̃* = 0.06 *T*_2_, enabling more than 10^2^ gates between correction cycles.

*Scaling*. We finally extend our analysis to a generic qudit spin *S*. We introduce as the unit of time , i.e., a few times the time required for a π pulse (*B*_1_ being the peak amplitude of the Gaussian pulse). Figure 3 shows that for reasonable values of *T*_2_ and even small *S*, we obtain a very large *T̃*/τ, roughly representing the number of operations which can be performed before we need to correct.

**Figure 3:**
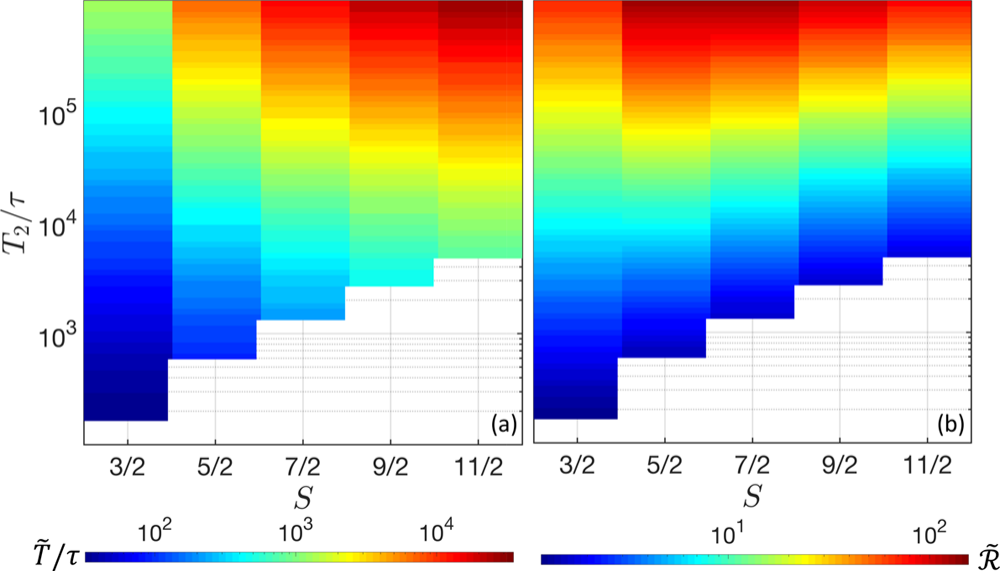
Optimal working point *T̃* (a) and gain  at the optimal working point (b) as a function of *T*_2_ and of the qudit spin *S* (instantaneous operations of the ancilla are assumed). Times are in units of the elementary gating time τ. A cutoff is applied to exclude data with . Therefore, the reported results correspond to very large final fidelities. For simplicity, we neglect small gating errors (which can be further reduced by chemically tailoring the molecular spectrum and designing the pulse shape).

In particular, this value increases with *S* (due to improved QEC) and *T*_2_.  shows a maximum at intermediate spin values, because the time needed to implement QEC increases approximately linearly with *S* (see the Supporting Information), thus marking a trade-off between the increase in the number of correctable errors (number of powers of *S*_*z*_ whose effect can be corrected) and the effect of dephasing during the correction procedure. Remarkably, our procedure yields large error reductions (large ) even with very large *T̃*/τ, i.e., if QEC is not frequently repeated. For instance, Figure 3 shows that even for *S* = 3/2 and *T*_2_/τ = 2 × 10^4^ we get  and *T̃*/τ ≈ 200.

In summary, we have shown that molecular nanomagnets, thanks to their spectrum characterized by many accessible levels, can be used to encode robust error-corrected qubits in single molecules. The single-object nature of the logical units yields several advantages, compared to standard multiqubit platforms:^24^ (i) It exploits the many levels in the Hilbert space as a resource, rather than considering them as a leakage source only. (ii) It largely reduces hardware overhead when building up a processor. (iii) It makes logical operations (especially two-qubit gates) easier to realize. In addition, as compared to other multilevel codes which are based on generic error models,^20,21^ our scheme is targeted to the major error source in the real system. These reasons make our route promising for the realization of a scalable quantum processor.^62−64^

We finally point out that the proposed scheme can be realized using a variety of magnetic molecules with large nuclear or electronic (effective) spin and a significant *D* to resolve all transitions by magnetic pulses. It could also be extended to a wider class of molecular systems, by exploiting the flexibility in the level structure to achieve stronger qubit protection. For instance, molecules with competing interactions are characterized by many low-spin low-energy multiplets,^65−72^ thus enabling the increase of the number of levels without having large Δ*m* between the states (thus reducing decoherence). Matrix elements between different multiplets can be chemically engineered by using ions with distinct *g* values, thus reducing the number of operations required by the code. As a result, more effective encodings could be found, involving potential protection against different classes of errors.

## Supporting Information Available

Details on Lindblad equation for pure dephasing, derivation of the code-words, description of the general QEC procedure and of its decomposition in terms of elementary pulses, numerical simulations, and refs (73 and 74) (PDF)
