# Supplementary material for: Molecular Nanomagnets as Qubits with Embedded Quantum-Error Correction
Source: J Phys Chem Lett. 2020 Sep 16;11(20):8610–5. doi: 10.1021/acs.jpclett.0c02213 (PMC8011924; doi:10.1021/acs.jpclett.0c02213)
Supplement: Supplementary file 1 — jz0c02213_si_001.pdf [file jz0c02213_si_001.pdf]

# Supporting information for: Molecular Nanomagnets as Qubits with Embedded Quantum-Error Correction

A. Chiesa,[†,‡,§] E. Macaluso,[†,§] F. Petiziol,[†,‡,§] S. Wimberger,[†,¶] P. Santini,[†,‡] and S. Carretta[*,†,‡]

†*Dipartimento di Scienze Matematiche, Fisiche e Informatiche, Università di Parma, I-43124 Parma, Italy*

‡*UdR Parma, INSTM, I-43124 Parma, Italy*

¶*INFN, Sezione di Milano Bicocca, Gruppo Collegato di Parma, Parma, Italy*

§*These authors contributed equally to this work*

E-mail: stefano.carretta@unipr.it

# 1 Quantum error correction (QEC) for spin dephasing

## 1.1 Description of pure spin dephasing

The qudit is subject to dephasing induced by fluctuations of the magnetic field produced, e.g., by the surrounding nuclei. Hence, we can describe the qudit-environment interaction by a coupling of the form $S_z \otimes \mathcal{B}$, where $\mathcal{B}$, is a suitable operator acting on the Hilbert space of the bath. Following standard derivations of master equations in the Born-Markov approximation,[S1] we find that the qudit's density matrix $\rho(t)$ evolves according to the Lindblad equation

$$\frac{d\rho(t)}{dt} = \gamma \left( S_z \rho(t) S_z - \frac{1}{2} \left\{ S_z^2, \rho(t) \right\} \right), \tag{S1}$$

with rate $\gamma = 2/T_2$. Adopting a quantum-error-correction perspective, it is convenient to write the solution of eq (S1) in Kraus form, $\rho(t) = \sum_{k=0}^{\infty} E_k \rho(0) E_k^\dagger$ with $\sum_k E_k^\dagger E_k = \mathbb{I}$. For

a spin $S$, the Kraus "error" operators are

$$E_k = \sqrt{\frac{(\gamma t)^k}{k!}} e^{-\gamma t S_z^2/2} S_z^k. \tag{S2}$$

These operators can be found from eq (S1) by first moving to the interaction frame with respect to the anti-commutator part appearing on the r.h.s.,

$$\rho(t) = e^{-\gamma t S_z^2/2} \rho_I(t) e^{-\gamma t S_z^2/2}, \tag{S3}$$

which produces the following equation for $\rho_I(t)$,

$$\frac{d\rho_I(t)}{dt} = \gamma S_z \rho_I(t) S_z. \tag{S4}$$

By formally integrating eq (S4) as a Dyson series from $t = 0$ to $t$, one then obtains

$$\rho_I(t) = \rho_I(0) + \gamma \int_0^t dt_1 S_z \rho_I(0) S_z + \frac{\gamma^2}{2!} \int_0^t dt_1 \int_0^t dt_2 S_z^2 \rho_I(0) S_z^2 + \dots \tag{S5}$$

Performing the integrals and reverting to Schrödinger picture we finally obtain

$$\rho(t) = \sum_{k=0}^{\infty} \frac{(\gamma t)^k}{k!} e^{-\gamma t S_z^2/2} S_z^k \rho(0) S_z^k e^{-\gamma t S_z^2/2}, \tag{S6}$$

and one can evince the Kraus operators given in eq (S2).

By expanding $\rho(t)$ over the eigenstates $\{|m\rangle\}$ of $S_z$, one finds that eq (S1) leaves the diagonal elements of $\rho$ unaltered, while it induces an exponential decay of coherences with the squared difference of eigenvalue of $S_z$:

$$\rho(t) = \sum_{m',m=-S}^{S} e^{-\gamma t (m-m')^2/2} \langle m| \rho(0) |m'\rangle |m\rangle \langle m'| . \tag{S7}$$

For correcting $\rho(t)$ to order $(\gamma t)^n$, one needs to correct error operators $E_k$ up to $k = n$. For this to be possible, Knill-Laflamme's quantum-error correction (QEC) conditions must be satisfied for these operators, i.e., for $0 \leq k, j \leq n$,

$$\langle 0_L| E_k^\dagger E_j |0_L\rangle = \langle 1_L| E_k^\dagger E_j |1_L\rangle , \tag{S8a}$$

$$\langle 0_L| E_k^\dagger E_j |1_L\rangle = 0. \tag{S8b}$$

One can verify that the conditions (S8) automatically hold if they are fulfilled for error operators $S_z^k$ with $0 \leq k \leq 2n$. Hence, to achieve precision $(\gamma t)^n$, we search for code words $|0_L\rangle$ and $|1_L\rangle$ such that

$$\langle a_L| S_z^k |b_L\rangle = \beta_k \delta_{a,b}, \quad 0 \leq k \leq 2n. \tag{S9}$$

Let us point out that the error operators (and hence Knill-Laflamme conditions) are related to powers of $S_z$ because the system eigenstates are also practically eigenstates of $S_z$ and

hence this is the system operator which couples to the environment. This can be ensured by choosing a significant magnetic field (along $z$) which sets the quantization axis. The whole scheme described in this work can be applied whenever this is the case.

## 1.2 Binomial code words

Inspired by bosonic binomial codes, introduced in Ref.,[S2] we define the following code words:

$$|0_L\rangle = \frac{1}{\sqrt{2^{2S-1}}} \sum_{\substack{k=1 \\ k \text{ odd}}}^{2S} \sqrt{\binom{2S}{k}} |k-S\rangle \,, \tag{S10a}$$

$$|1_L\rangle = \frac{1}{\sqrt{2^{2S-1}}} \sum_{\substack{k=0 \\ k \text{ even}}}^{2S} \sqrt{\binom{2S}{k}} |k-S\rangle \,. \tag{S10b}$$

We now show that the states (S10) fulfill the KL conditions of eq (S9). It is evident that $\langle 0_L| S_z^\ell |1_L\rangle = 0$ since $|0_L\rangle$ and $|1_L\rangle$ are superpositions of different sets of states which are not mixed by dephasing. Focusing on expectation values of $S_z^\ell$ over the same code word, we get

$$\langle 1_L| S_z^\ell |1_L\rangle - \langle 0_L| S_z^\ell |0_L\rangle = \frac{1}{2^{2S-1}} \sum_{k=0}^{2S} (-1)^k \binom{2S}{k} \langle k-S| S_z^\ell |k-S\rangle \,, \tag{S11a}$$

$$= \frac{1}{2^{2S-1}} \sum_{k=0}^{2S} (-1)^k \binom{2S}{k} (k-S)^\ell. \tag{S11b}$$

Using the binomial expansion,

$$(1+x)^{2S} = \sum_{m=0}^{2S} \binom{2S}{m} x^m, \tag{S12}$$

and assuming $\ell < 2S$, one finds that

$$\begin{aligned} \sum_{m=0}^{2S} \binom{2S}{m} (-1)^m (m-S)^\ell &= \left[ \left( x \frac{\mathrm{d}}{\mathrm{d}x} \right)^\ell \sum_{m=0}^{2S} \binom{2S}{m} (-1)^m x^{m-S} \right]_{x=1} \\ &= \left[ \left( x \frac{\mathrm{d}}{\mathrm{d}x} \right)^\ell \frac{1}{x^S} (1-x)^{2S} \right]_{x=1} = 0. \end{aligned} \tag{S13}$$

Hence, the validity of the code words (S10) is verified. In particular, eqs (S9) are satisfied for powers of $S_z$ up to exponent $2S-1$ included. Since Knill-Laflamme conditions (S9) must be fulfilled for powers of $S_z$ up to $2n$ included for attaining correction up to order $(\gamma t)^n$, one can see that perturbative order $n$ requires at least spin $S = n + 1/2$. Moreover, since two levels ($2n$) are needed for each perturbative order to be corrected ($n$), one can also see that

an integer spin $S_{int}$ provides the same correction order as the half-integer spin $S_{int} - 1/2$. This justifies our attention to half-integer spins in the main text. In conclusion, a spin $S$ is sufficient for correcting $\rho(t)$ up to order $(\gamma t)^{\lfloor S \rfloor}$. Here, $\lfloor x \rfloor$ indicates the largest integer smaller than $x$.

## 2 Ideal quantum error correction procedure

We here formulate an ideal detection+recovery procedure, following the general QEC prescriptions of Knill and Laflamme.[S3] The error spaces $\mathcal{V}_\ell$, with $\ell = \mathbb{0}, \mathbb{1}$, is defined as the space spanned by the (normalized) set of states $S_z^k \left|\ell_L\right\rangle$, $0 \leq k \leq \lfloor S \rfloor$ ($\lfloor S \rfloor$ being the largest integer smaller than $S$), which are produced by the action of the error operators on the code word $\left|\ell_L\right\rangle$. In formulae,

$$\mathcal{V}_\ell = \mathrm{span}\left\{ \frac{S_z \left|\ell_L\right\rangle}{\|S_z \left|\ell_L\right\rangle\|}, \ldots, \frac{S_z^{\lfloor S \rfloor} \left|\ell_L\right\rangle}{\left\|S_z^{\lfloor S \rfloor} \left|\ell_L\right\rangle\right\|} \right\}. \tag{S14}$$

The first requirement for performing error correction is the ability to detect and distinguish different errors without corrupting the quantum information encoded (i.e., superpositions of $\left|0_L\right\rangle$ and $\left|1_L\right\rangle$). Since for pure-dephasing errors the vectors $S_z^k \left|\ell_L\right\rangle$ do not form an orthonormal set, the first step is to fix an orthonormal basis in the error spaces. Fulfillment of Knill-Laflamme's conditions, eq (S9), guarantees that there exists a unitary $\mathcal{U}$ such that

$$\mathcal{U} S_z^k \left|0_L\right\rangle = S_z^k \left|1_L\right\rangle . \tag{S15}$$

This implies that errors on the code words preserve logical superpositions. Once a basis $\{\left|E_{\mathbb{0}}^k\right\rangle\}_{k=0,\ldots,\lfloor S \rfloor}$ of error words for the error space $\mathcal{V}_{\mathbb{0}}$ is chosen, the basis $\{\left|E_{\mathbb{1}}^k\right\rangle\}_{k=0,\ldots,\lfloor S \rfloor}$ for $\mathcal{V}_{\mathbb{1}}$ must satisfy $\mathcal{U}\left|E_{\mathbb{0}}^k\right\rangle = \left|E_{\mathbb{1}}^k\right\rangle$. Keeping this in mind, we take as a basis the set of vectors obtained from a Gram-Schmidt orthonormalisation of states $S_z^k \left|\ell_L\right\rangle$. The error correction operation $\mathcal{O}_R$ is then defined by the collection of operations $\{O_k P_k\}_{k=0,\ldots,\lfloor S \rfloor}$, where $O_k$ and $P_k$ are such that

$$O_k \left|E_\ell^k\right\rangle = \left|\ell_L\right\rangle , \tag{S16a}$$

$$P_k = \left(\left|E_{\mathbb{0}}^k\right\rangle \left\langle E_{\mathbb{0}}^k\right| + \left|E_{\mathbb{1}}^k\right\rangle \left\langle E_{\mathbb{1}}^k\right|\right) . \tag{S16b}$$

In other words, the error correction is decomposed into a two-step process involving a measurement which projects into one of the different two-dimensional subspaces, $P_k = \left|E_{\mathbb{0}}^k\right\rangle \left\langle E_{\mathbb{0}}^k\right| + \left|E_{\mathbb{1}}^k\right\rangle \left\langle E_{\mathbb{1}}^k\right|$, followed by the corresponding restoring of the logical state by $O_k$.

We now show that this procedure, for an encoding operated on a spin $S$, guarantees recovery to unit fidelity up to order $(\gamma t)^{\lfloor S \rfloor}$. Indeed, let the initial state be $\rho(0) \equiv \rho_0 = \left|\psi_L\right\rangle \left\langle\psi_L\right|$ with $\left|\psi_L\right\rangle = \sqrt{p}\left|0_L\right\rangle + \sqrt{1-p}e^{-i\phi}\left|1_L\right\rangle$, which dephases according to the eq (S7).

The fidelity of the QEC procedure performed instantaneously after a time $t$ is

$$\begin{aligned}\mathcal{F}_S^2(t) &= \langle\psi_L|\, \mathcal{O}_R\left[\rho(t)\right]|\psi_L\rangle\,,\\ &= \langle\psi_L|\sum_{k=0}^{\lfloor S\rfloor} O_k P_k \rho(t) P_k O_k^\dagger\,|\psi_L\rangle\,. \end{aligned}\tag{S17}$$

Inserting eq (S7) into eq (S17) and using eqs (S16), we find

$$\mathcal{F}_S^2(t) = \sum_{m,m'=-S}^{S} e^{-\gamma t(m-m')^2/2}\rho_0^{(m,m')}\sum_{\ell,\ell'=0,1}\sum_{k=0}^{\lfloor S\rfloor}\langle E_\ell^k|m\rangle\,\langle m'|E_{\ell'}^k\rangle\,|\ell_L\rangle\,\langle\ell'_L|\,. \tag{S18}$$

After changing summation variables according to $\mu = m+S$, $\mu' = m'+S$, we break these summations depending on the parity of $\mu$ and $\mu'$. After some manipulations, this eventually leads to the expression

$$\mathcal{F}_S^2(t) = 1-2p(1-p)[1-\xi_S(t)], \tag{S19}$$

where $\xi_S(t)$ is defined as

$$\xi_S(t) = \frac{1}{2^{2S-1}}\sum_{\substack{\mu=0\\ \mu\text{ even}}}^{2S}\sum_{\substack{\mu'=0\\ \mu'\text{ odd}}}^{2S} e^{-\gamma t(\mu-\mu')^2/2}\sqrt{\binom{2S}{\mu}\binom{2S}{\mu'}}\,\langle\mu'-S|\,\mathcal{U}\,|\mu-S\rangle\,. \tag{S20}$$

In deriving eq (S19) and (S20), we have used that $|0_L\rangle$ [$|1_L\rangle$] has components on states $|\mu-S\rangle$ with even [odd] $\mu$ only (see eqs (S10)). We have also used the explicit expressions for $\langle\mu-S|\,\rho_0\,|\mu'-S\rangle$, the fact that $\sum_{k=0}^{\lfloor S\rfloor}|E_\ell^k\rangle\,\langle E_\ell^k|$ acts as the identity operator on $\mathcal{V}_\ell$, the definition (S15) of $\mathcal{U}$, and we have exploited that $\mathcal{U}$ is real orthogonal, implying that $\langle\mu-S|\,\mathcal{U}^\dagger\,|\mu'-S\rangle = \langle\mu'-S|\,\mathcal{U}\,|\mu-S\rangle$. Furthermore, we have used that $\sum_{\substack{k=0\\ k\text{ even/odd}}}^{2S}\binom{2S}{k} = 2^{2S-1}$. Note that the fidelity for a freely-dephasing spin 1/2 can be expressed as well in the form of eq (S19) with $\xi_{1/2}(t) = e^{-\gamma t/2}$. Expanding $e^{-\gamma t(\mu-\mu')^2/2}$ in powers of $\gamma t$ and using the binomial theorem (S12), we can write

$$e^{-\gamma t(\mu-\mu')^2/2} = \sum_{k=0}^{\infty}\frac{1}{k!}\left(\frac{-\gamma t}{2}\right)^k(\mu-\mu')^{2k}, \tag{S21a}$$

$$= \sum_{k=0}^{\infty}\frac{1}{k!}\left(\frac{-\gamma t}{2}\right)^k\sum_{j=0}^{2k}\binom{2k}{j}(\mu-S)^j(-\mu'+S)^{2k-j}. \tag{S21b}$$

Inserting (S21b) into (S20) and recalling that

$$S_z^k\,|0_L/1_L\rangle = \frac{1}{\sqrt{2^{2S-1}}}\sum_{\substack{\mu=0\\ \mu\text{ odd/even}}}^{2S}\sqrt{\binom{2S}{\mu}}(\mu-S)^k\,|\mu-S\rangle\,, \tag{S22}$$

Equation (S20) finally becomes

$$\xi_S(t) = \sum_{k=0}^{\infty} \frac{(-\gamma t/2)^k}{k!} \sum_{j=0}^{2k} \binom{2k}{j} (-1)^j \langle 1_L| S_z^j \mathcal{U} S_z^{2k-j} |0_L\rangle \,. \tag{S23}$$

Now, considering a spin $S$, Knill-Laflamme's QEC conditions (S9) can be satisfied for powers of $S_z$ up to $S_z^{2\lfloor S\rfloor}$. Then, because of eq (S15), for $1 \leq k \leq \lfloor S\rfloor$ it holds that $\langle 1_L| S_z^j \mathcal{U} S_z^{2k-j} |0_L\rangle = \langle 1_L| S_z^{2k} |1_L\rangle$. This, together with the fact that $\sum_{j=0}^{2k} \binom{2k}{j} (-1)^j = 0$, gives

$$\xi_S(t) = 1 + \sum_{k=\lceil S\rceil}^{\infty} \frac{1}{k!} \left(-\frac{\gamma t}{2}\right)^k \sum_{j=1}^{2k} \binom{2k}{j} (-1)^j \langle 1_L| S_z^j \mathcal{U} S_z^{2k-j} |0_L\rangle \,. \tag{S24}$$

Therefore, it holds that $\xi_S(t) = 1 + \mathcal{O}[(\gamma t)^{\lceil S\rceil}]$ and, resuming eq (S20), the fidelity post recovery is:

$$\mathcal{F}_S^2(t) = 1 - 2p(1-p)\mathcal{O}[(\gamma t)^{\lceil S\rceil}], \tag{S25}$$

confirming the effectiveness of the quantum error correction procedure, increasing with $S$. From eq (S24) it is also evident that $\xi_S(0) = 1$ while from eq (S20) one can seen that $\xi_S(t) \overset{t\to+\infty}{\longrightarrow} 1$.

As a last point, let us produce a geometric interpretation of the efficiency of the error correction. The fidelity of the recovery, eq (S19), can also be rewritten like

$$\mathcal{F}_S^2(t) = p^2 + (1-p)^2 - 2p(1-p)\cos(\theta_S) \tag{S26}$$

with, e.g., $\theta_S = \pi - \arccos \xi_S(t)$. Hence, the fidelity can be visualised as the third side of a triangle whose other two sides have length $p$ and $1-p$ and identify an angle $\theta_S$. At the initial time, $\theta_S$ equals $\pi$, the triangle collapses to a line and the fidelity is equal to one. Asymptotically, dephasing makes $\theta$ shrink towards the value $\pi/2$, forming a right triangle. The effectiveness of the QEC when $S$ increases can then be interpreted as a slowing down of the rate of variation of $\theta_S$.

# 3 Explicit sequence of pulses for quantum error correction in the real system

In this section, we discuss how the procedure described in Sec. 2 can be operated explicitly in molecular magnets. The major challenge is that of designing a sequence of pulses which implements the measurement and recovery operation of eq (S16). The general procedure described below is represented schematically in Fig. S2, which extends Fig. 2 of the main text to the case of a general spin $S$. For an easier interpretation of both figures, a detailed description of a sub-panel of Fig. 2 is furnished in Fig. S1.

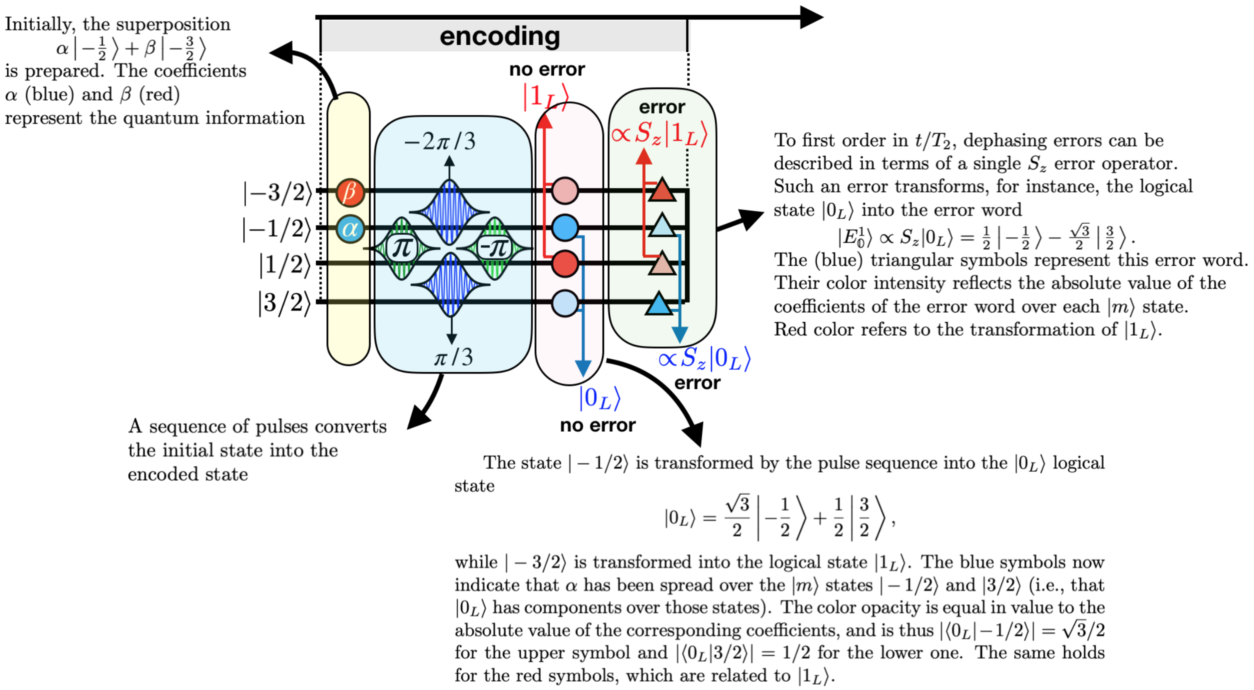


Figure S1: Details about different graphical elements of Fig. 2 of the main text, by focusing on the "encoding" sub-panel.

## 3.1 Error detection

The first step concerns the syndrome extraction—that is, the measurement which implements the projectors $P_k$ of eq (S16b) and detects which error (including the no-error case) has occurred. Our measurement apparatus exploits excitations of a spin 1/2 electronic ancilla, conditioned on the state of the qudit. In order to use this mechanism to distinguish different error words $|E^k_\ell\rangle$, we first need to rotate each $|E^k_\ell\rangle$ (in both error spaces $\ell = \mathbb{0}, \mathbb{1}$) into a spin projection. Let us indicate with $|m^k_\ell\rangle$ the spin projection associated with the error word $|E^k_\ell\rangle$, corresponding to the $k$-th error and the logical state $\ell = \mathbb{0}$ or $\ell = \mathbb{1}$. Specifically, we look for a rotation $U$, such that $U\,|E^k_\ell\rangle = |m^k_\ell\rangle$. This is represented in the panel "Detection" of Fig. S2. Once $U$ has been applied, we use two microwave pulses resonant with transitions $|m^k_\ell\rangle \otimes \downarrow \to |m^k_\ell\rangle \otimes \uparrow$ ($\ell = \mathbb{0}, \mathbb{1}$) to implement the conditional excitation of the ancilla represented by the transformation $\mathbb{E}_k = \left(|m^k_{\mathbb{0}}\rangle\langle m^k_{\mathbb{0}}| + |m^k_{\mathbb{1}}\rangle\langle m^k_{\mathbb{1}}|\right) \otimes |\uparrow\rangle\langle\downarrow|$. Note that the excitation energy of the ancilla depends on the state $|m^k_\ell\rangle$ of the qudit through the ancilla-qudit coupling $\Gamma$ in Hamiltonian (1) of the main text. This allows one to perform an indirect non-demolition measurement of $S^k_z$ through a measurement of $\sigma^A_z$ of the ancilla. In particular, for each error a conditional excitation is performed, followed by a measurement of the ancilla: if the ancilla is found in the excited state, then the error has been detected and the recovery operation starts. Otherwise, the sequence of conditional excitations and ancilla measurements continues in order to check all possible errors one by one until a positive outcome (ancilla $\uparrow$) is found. The order in which different errors are checked is chosen such that most probable errors, corresponding to lower powers of $S_z$, are checked first. In this way, the overall number of measurements which is typically performed remains small as

compared to the number of possible errors.
One may be tempted to choose $|E_\ell^k\rangle$ to match the basis $|m_\ell^k\rangle$ from the start in order to avoid performing the rotation: this, however, would produce a corruption of the quantum information since it is not true that $|m_{\mathbb{1}}^k\rangle = \mathcal{U}\,|m_{\mathbb{0}}^k\rangle$, for $\mathcal{U}$ defined in (S15). Also, one must ascertain that the extra step $U$, combined with the measurement, still does not corrupt the quantum information.
Depending on the outcome of the measurement of $S_z$, a recovery process restores the corresponding code word in each logical space. The projection+recovery procedure of eq (S16) is then in practice implemented as $O_k P_k = \tilde{O}_k \tilde{P}_k U$ with

$$\tilde{O}_k\,|m_\ell^k\rangle = |\ell_L\rangle\,, \tag{S27a}$$

$$\tilde{P}_k = \sum_{\ell=\mathbb{0},\mathbb{1}} |m_\ell^k\rangle\,\langle m_\ell^k|\,. \tag{S27b}$$

The next step is then to describe how to implement these two transformations in practice.

## 3.2 Determination of the transformation $U$

For realizing $U$ explicitly, we resort to a two-step procedure. First, we perform a sequence of pulses which shifts the support of $|0_L\rangle$ (over the eigenstates of $S_z$) to the $\lceil S\rceil$ eigenstates of $S_z$ with lower eigenvalues and the support of $|1_L\rangle$ to the $\lceil S\rceil$ states with larger eigenvalues (always maintaining the same coefficients), see Fig. S2. Let us call these "shifted" error words $|e_\ell^k\rangle$. For clarity, let us stress that these states are not a redefinition of the code words, but just a transformation which is useful to implement the detection procedure. This allows us to manipulate, in the next step, the two error subspaces independently and in parallel in a convenient way. At this point, we realize, over each subspace, the unitary transformation indicated by $M_{\rm rot}$ in Fig. S2 which maps each shifted error word $|e_\ell^k\rangle$ into an $|m_\ell^k\rangle$ state. Determining a sequence of pulses which implements this full unitarity is in general not obvious. For this reason, we develop a general "compiling" procedure, which decomposes $M_{\rm rot}$ into a sequence of $|\Delta m| = 1$ state transfers. The resulting sequence (although not necessary the best possible one in terms of the number of operations), can be systematically applied to general-spin problems and it can be used as a starting point for further case-by-case optimization. The procedure works as follows. As a preliminary remark, let us point out that, since all states involved have real components on the $|m\rangle$ basis, $M_{\rm rot}$ can be chosen as a real orthogonal matrix. Specifically, for each subspace $\ell$, the transformation $M_{\rm rot}$ can be chosen as $M_{\rm rot} = \sum_{\ell=\mathbb{0}}^{\mathbb{1}} \sum_{k=0}^{\lfloor S\rfloor} |m_\ell^k\rangle\,\langle e_\ell^k| = M_{\rm rot}^{\mathbb{0}} + M_{\rm rot}^{\mathbb{1}}$, and it contains in general non-zero matrix elements over all couples of spin projections. For instance, we choose

$$M_{\rm rot}^{\mathbb{0}} = \sum_{k=0}^{\lfloor S\rfloor} |-1/2-k\rangle\,\langle e_{\mathbb{0}}^k|\,;\;\; M_{\rm rot}^{\mathbb{1}} = \sum_{k=0}^{\lfloor S\rfloor} |S-k\rangle\,\langle e_{\mathbb{1}}^k|\,. \tag{S28}$$

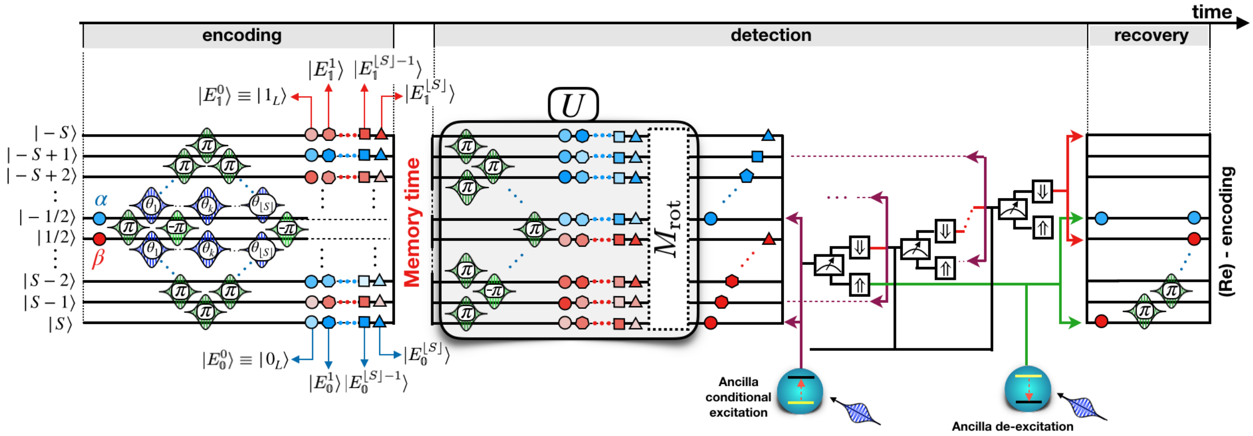


Figure S2: Graphical representation of the sequence of pulses realizing the quantum error correction procedure for a generic spin $S$. The schematics used is the same as in Fig. 2(a) of the article. The encoding step is characterized by $|-1/2\rangle \rightarrow |-1/2-1\rangle$ and $|1/2\rangle \rightarrow |1/2+1\rangle$ transitions with $\sin^2\frac{\vartheta_k}{2} = \binom{2S}{k-1}/[2^{2S-1} - \sum_{j=0}^{k-2}\binom{2S}{j}]$ (blue), while all the others are $\pm\pi$ pulses (green) with alternating signs in the central $|1/2\rangle \leftrightarrow |-1/2\rangle$ transition. After the memory time, a sequence of pulses moves the components of each code-words close to each other (without changing their signs, possibly adding full Rabi flops of the ancilla to correct it, depending on $S$). A subsequent sequence implements the unitary transformation $M_{\rm rot}$, whose decomposition into elementary $R_y^{mm'}(\vartheta)$ operations is described in detail in the text. Consecutive conditional excitations of the ancilla (depending on state $|m\rangle$ of the qudit) allow one to detect errors by non-demolition measurement. Finally, depending on the ancilla measurement outcome $\uparrow / \downarrow$ (here we indicate the case $\sigma_z^A = \uparrow$ for $k=0$), a different sequence of $\pi$ pulses is applied to restore the initial superposition state $\alpha\,|-1/2\rangle + \beta\,|1/2\rangle$ and the encoding procedure is repeated. In case of $\downarrow$ ancilla measurement outcome the conditional excitation is repeated until $k = \lfloor S \rfloor$ or the first positive $\sigma_z^A$ is obtained. The details of the reported sequence of pulses hold for even $S+1/2$. Very small modifications are needed for the case of odd $S+1/2$ (see, e.g., the description of the sequence for $S=5/2$ below).

For a spin $S = 7/2$, our choice of $M_{\rm rot}$ thus maps

$$|e^0_{\mathbb{0}}\rangle \to |{-1/2}\rangle\,, \quad |e^0_{\mathbb{1}}\rangle \to |7/2\rangle\,, \tag{S29a}$$
$$|e^1_{\mathbb{0}}\rangle \to |{-3/2}\rangle\,, \quad |e^1_{\mathbb{1}}\rangle \to |5/2\rangle\,, \tag{S29b}$$
$$|e^2_{\mathbb{0}}\rangle \to |{-5/2}\rangle\,, \quad |e^2_{\mathbb{1}}\rangle \to |3/2\rangle\,, \tag{S29c}$$
$$|e^3_{\mathbb{0}}\rangle \to |{-7/2}\rangle\,, \quad |e^3_{\mathbb{1}}\rangle \to |1/2\rangle\,. \tag{S29d}$$

Then, indicating with $|m\rangle$ and $|m'\rangle$ two generic spin projections, we perform a decomposition of $M^{\mathbb{0}}_{\rm rot}$ and $M^{\mathbb{1}}_{\rm rot}$ into (real) planar rotations

$$V_{\mathbb{0}}^{-1/2-m,-1/2-m'} = \cos\frac{\theta}{2}[|m\rangle\langle m| + |m'\rangle\langle m'|] + \sin\frac{\theta}{2}[|m\rangle\langle m'| - |m'\rangle\langle m|] + \sum_{n\neq m,m'} |n\rangle\langle n|\,, \tag{S30}$$

$$V_{\mathbb{1}}^{S-m,S-m'} = \cos\frac{\theta}{2}[|m\rangle\langle m| + |m'\rangle\langle m'|] + \sin\frac{\theta}{2}[|m\rangle\langle m'| - |m'\rangle\langle m|] + \sum_{n\neq m,m'} |n\rangle\langle n|\,, \tag{S31}$$

with $m' > m = -S, ..., S$, following the algorithm in Ref.[S4] This step returns a sequence of $\lceil S\rceil(\lceil S\rceil - 1)/2$ unitarities for each error subspace, describing single two-state transitions (though with no restrictions on $\Delta m$), which are generated by "generalized" $\sigma_y$ matrices. Specifically, indicating with $N = \lceil S\rceil$ and using the shortcut $V^{n,l}_\ell \equiv V^{n,l}_\ell(\theta_{n,l})$, one obtains a decomposition of the form

$$M^\ell_{\rm rot} = V^{0,1}_\ell V^{0,2}_\ell V^{1,2}_\ell \dots V^{N-4,N-3}_\ell V^{0,N-2}_\ell \dots V^{N-3,N-2}_\ell V^{0,N-1}_\ell \dots V^{N-3,N-1}_\ell V^{N-2,N-1}_\ell. \tag{S32}$$

Then, each transition involving states with $\Delta m > 1$ is further decomposed as a sequence of $\Delta m - 1$ transfers. This is done by observing that

$$V^{n,l}_\ell(\theta) = V^{j,l}_\ell(\pi) V^{n,j}_\ell(\theta) V^{j,l}_\ell(-\pi), \tag{S33}$$

for any $n < j < l$. Then, this decomposition is iterated until a sequence of $\Delta m = 1$ transitions is obtained, i.e.

$$\begin{aligned} V^{n,l}_\ell(\theta) =& V^{l-1,l}_\ell(\pi) V^{n,l-1}_\ell(\theta) V^{l-1,l}_\ell(-\pi) = \\ =& V^{l-1,l}_\ell(\pi) V^{l-2,l-1}_\ell(\pi) V^{n,l-2}_\ell(\theta) \cdot V^{l-2,l-1}_\ell(-\pi) V^{l-1,l}_\ell(-\pi) = \\ =& V^{l-1,l}_\ell(\pi) V^{l-2,l-1}_\ell(\pi) \cdot \ldots \cdot V^{n+1,n+2}_\ell(\pi) V^{n,n+1}_\ell(\theta) V^{n+1,n+2}_\ell(-\pi) \cdot \\ & \ldots \cdot V^{l-2,l-1}_\ell(-\pi) V^{l-1,l}_\ell(-\pi). \end{aligned} \tag{S34}$$

Now, let us notice that, in the combination of rotations appearing in eq (S32), many of the $\pi$-pulses given by eq (S34) simplify. In particular, for a fixed second index $n$, we have that

$V_{0,n-1}\dots V_{n-2,n-1}$ can be written as

$$V_\ell^{0,n-1}(\theta_{0,n-1})\dots V_\ell^{n-2,n-1}(\theta_{n-2,n-1}) = \left(\overleftarrow{\prod}_{j=1}^{n-2} V_\ell^{j,j+1}(\pi)\right) V_\ell^{0,1}(\theta_{0,n-1}) \left(\overrightarrow{\prod}_{j=1}^{n-2} V_\ell^{j,j+1}(-\pi+\theta_{j,n-1})\right), \quad \text{(S35)}$$

where $\overleftarrow{\prod}$ and $\overrightarrow{\prod}$ indicate that, in the product of matrices, the index $j$ increases from right to left and vice versa. Therefore, for each "second" index $n$ of $V_\ell^{x,n}$ one obtains a product of $2n-3$ rotations. Summing over $n$ from $n=1$ to $n=N-1$ (see eq (S32)), we find that the total number of pulses for realizing $M_{\text{rot}}^k$ is $(N-1)^2$. The sequences for $\ell = \mathbb{0}$ and $\ell = \mathbb{1}$, since they involve independent set of states, can be performed in parallel. Hence, the scaling with $S$ of the number of pulses required to implement the full basis rotation is quadratic. This is quite remarkable given that a quadratic scaling was found as well for the decomposition without restrictions on $\Delta m$ (see the paragraph after eq (S29)).

## 3.3 Recovery

Once the ancilla has been measured, the qudit state is a superposition $\alpha\,|m\rangle + \beta\,|m'\rangle$ with $m$ and $m'$ depending on the result of the measurement. The recovery can then be performed by reordering the populations such that this state is transferred to $\alpha\,|{-1/2}\rangle + \beta\,|1/2\rangle$, and then operating the encoding procedure from scratch.

## 3.4 Scaling of the number of pulses with $S$

To summarize, we estimate the total number of pulses required to implement the full QEC code. Referring to Fig. S2, we note that the $d-1$ pulses are needed for the encoding, $d/2$ to "shift" the code-words before $M_{\text{rot}}$, $(d/2-1)^2$ to implement $M_{\text{rot}}$, $d/2-1$ for the recovery. Finally, a maximum number of $d/2$ conditional excitations of the ancilla + 1 de-excitation precedes the repetition of the encoding step. Recalling that $d = 2S+1$, we get $S^2+6S+3/4$ pulses to implement the code.

If however we consider the duration of the full pulse sequence, $T_{\text{QEC}}$, we obtain an approximately linear scaling as a function of the qudit spin $S$ (see Fig. S3). The explanation of this quasi-linear behaviour, despite the quadratic scaling of the number of operations described in Sec. 3.2, is attributed to that fact that (i) some of the operations (pulses) can be performed in parallel and (ii) for larger spins the matrix elements for a given transition increase in strength, thus reducing the time of each operation.

# 4 Impact of the duration of the QEC code

To actually assess the performance of any QEC scheme, we must consider the time needed to practically implement the QEC operations. For this reason, we have performed a simulation which takes into account the finite-time duration of each coherent operation, but assumes such operations are perfect, i.e., with unit fidelity. Indeed, any leakage arising during the

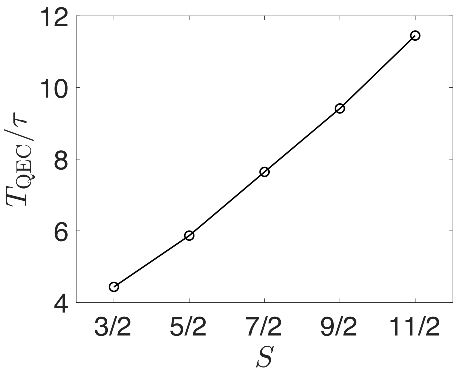


Figure S3: Total time required for implementing the QEC code described above, in units of $\tau$, i.e. the duration of a $\pi$ pulse for a spin 1/2 system. Measurements and excitations of the ancilla are assumed to be instantaneous.

implementation of coherent transformations is system specific and can be engineered by careful designing the molecular spectrum or the pulse shape. To eliminate leakage, we have divided the time evolution into steps corresponding to different pulses. In each step, we have numerically solved the equation of motion

$$\frac{d\rho}{dt} = -i[H_p, \rho] + \frac{1}{T_2}\left[2S_z\rho S_z - S_z^2\rho - \rho S_z^2\right]. \tag{S36}$$

where $H_p^{m,m+1} = \Omega\left(i\left|m\right\rangle\left\langle m+1\right| - i\left|m+1\right\rangle\left\langle m\right|\right)/2$ with $\Omega$ the Rabi frequency of $|m\rangle \rightarrow |m+1\rangle$ transition. This is equivalent to use circularly polarized pulses to induce transitions only between the examined couple of levels (thus eliminating leakage) and then move to the interaction picture to eliminate the time-dependence in $H_p$.

# 5 More details on S=3/2 simulations

Simulations on the nuclear $S = 3/2$ $^{63}$Cu qudit in the $(PPh_4)_2[Cu(mnt)_2]$ complex[S5] have been performed using circularly polarized Gaussian pulses of peak amplitude $B_1 = 50$ G, yielding $\tau = 875$ ns and Rabi period for the $|-1/2\rangle \rightarrow |1/2\rangle$ transition $\tau/\left\langle 1/2\right| S_x \left|-1/2\right\rangle =$ 437 ns. This is smaller than $6\hbar\sqrt{\pi/2}/g_\perp B_1$ due to slight mixing ($\sim 2\%$) between electronic and nuclear excitations.
We report below the time evolution of the diagonal elements of $\rho$ subject to the sequence of pulses implementing the QEC code for the $k = 1$ (panels a and b for ancilla $\uparrow$ and ancilla $\downarrow$ subspaces, respectively) and $k = 0$ (c) cases.

The repetition of the error-correction cycles yields an enhanced $T_2$ compared to the uncorrected $S = 1/2$ system, as shown in Fig. S5 below. In particular, by using a repetition cycle corresponding to the optimal working point $\tilde{T}$ (see main text) we obtain an effective $T_2^{\text{eff}}$ 3 times larger than the uncorrected case.

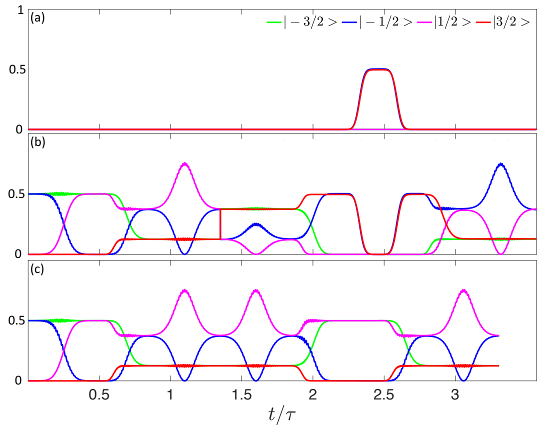


Figure S4: Diagonal elements of the density matrix for the simulations reported in Fig. 3 (main text) on the nuclear $S = 3/2$ system of Ref.[S5] in the case $k = 1$ (a,b) for the ancilla $\uparrow$ (a) and ancilla $\downarrow$ (b) subspaces and $k = 0$ (c).

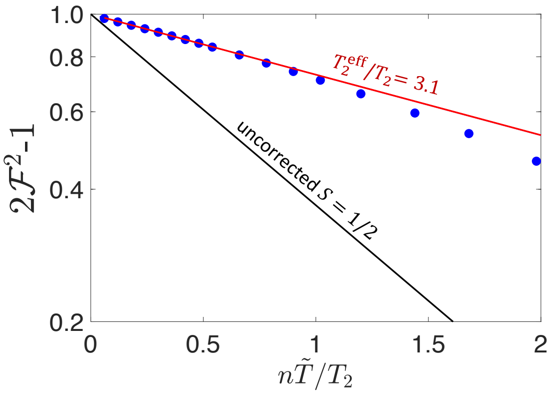


Figure S5: Simulated fidelity of the $S = 3/2$ qudit system reported in the main text (circles) after $n$ QEC cycles, repeated at time intervals $\tilde{T}$ determined from the maximum of the gain $\mathcal{R}$. Compared to a spin 1/2 uncorrected qubit, characterized by $2\mathcal{F}^2 - 1 = e^{-t/T_2}$, we obtain an enhancement of $T_2$ of a factor 3.1 (red line).

# References


(S1) Breuer, H.-P.; Petruccione, F. *The theory of open quantum systems*, Oxford University Press: Oxford, 2007.

(S2) Michael, M. H.; Silveri, M.; Brierley, R. T.; Albert, V. V.; Salmilehto, J.; Jiang, L.; Girvin, S. M. New Class of Quantum Error-Correcting Codes for a Bosonic Mode, *Phys. Rev. X* **2016**, *6*, 031006.

(S3) Knill, E.; Laflamme, R. Theory of Quantum Error-Correcting Codes, *Phys. Rev. A* **1997**, *55*, 900.

(S4) D'Alessandro, D. *Introduction to Quantum Control and Dynamics*, Chapman & Hall/CRC Applied Mathematics & Non-linear Science, CRC Press: 2007.

(S5) Bader, K.; Dengler, D.; Lenz, S.; Endeward, B.; Jiang, S.-D.; Neugebauer, P.; van Slageren, J. Room temperature quantum coherence in a potential molecular qubit, *Nat. Commun.* **2014**, *5*, 5304.
